# Assessment of Drivers of Antimicrobial Use and Resistance in Poultry and Domestic Pig Farming in the Msimbazi River Basin in Tanzania

**DOI:** 10.3390/antibiotics9120838

**Published:** 2020-11-24

**Authors:** Zuhura I. Kimera, Gasto Frumence, Leonard E. G. Mboera, Mark Rweyemamu, Stephen E. Mshana, Mecky I. N. Matee

**Affiliations:** 1 Muhimbili University of Health and Allied Sciences, P.O. Box 65001, 11103 Dar es Salaam, Tanzania; frumencegasto@yahoo.co.uk (G.F.); mateemecky@yahoo.com (M.I.N.M.); 2 Ministry of Livestock and Fisheries, Mtumba Area, P.O. Box 2182, 40487 Dodoma, Tanzania; 3 SACIDS Africa Centre of Excellence for Infectious Diseases, Sokoine University of Agriculture, P.O. Box 3297, 67125 Morogoro, Tanzania; lmboera@gmail.com (L.E.G.M.); mark.rweyemamu@btinternet.com (M.R.); 4 Catholic University of Health and Allied Sciences, P.O. Box 1464, 33109 Mwanza, Tanzania; stephen72mshana@gmail.com

**Keywords:** antimicrobial use, antimicrobial resistance, Msimbazi basin, poultry and domestic pig farmers, environment

## Abstract

Uncontrolled use of drugs both in humans and animals coupled with environmental contamination exacerbate the development and spread of antimicrobial resistance. This paper assessed the drivers of antimicrobial use and resistance in poultry and domestic pig farming and the environment. Questionnaires, in-depth interviews, and focus group discussions (FGDs) were used to collect information regarding demographic characteristics, knowledge, practices, attitudes, and perceptions of the drivers of antimicrobial use and resistance in animal farming and the environment. We found a higher proportion of usage of veterinary antimicrobials for prophylactic purposes (87.6%) in animal farming, than for therapeutic purposes (80.5%). The degree of farming experience was significantly (*p* < 0.05) related to the knowledge on the source of antimicrobial use, methods used in disease diagnosis, access to veterinary services, stocking of antimicrobials at home, and presence of agriculture activities that involve the use of manure. Uncontrolled disposal of wastes from households, disposal of human and veterinary drugs, and weak implementation of the legal framework was identified as the major contributors to the environment. The high usage of veterinary antimicrobials and the environmental contamination identified requires multisectoral interventions, as well as a review of government strategies, policies, and regulations on antimicrobial use.

## 1. Introduction

In Tanzania, the increased demand for short-cycle animal stocks such as poultry and domestic pigs has led to intensive animal production of these animals [1,2,3,4]. The poultry and domestic pigs are in most cases managed by women as essential elements for their income and empowerment [1,5,6]. According to national projections, the annual chicken meat and domestic pig production was expected to increase from 130,000 tonnes in 2017 to 465,600 tonnes in 2020 and from 22,000 in 2017 to 37,200 tons in 2022, respectively [7]. This increase has been attributed to a number of factors including increasing urbanization rate and increased trade of live animals and animal products [4].

The increasing animal production has been associated with several challenges [8,9,10] including a high number of animals being confined to poor quality shelters with limited space [1,11], overstretched veterinary extension services and lack of implementation of disease control strategies [12,13,14]. Expectedly, the frequency and magnitude of infectious diseases are high [15], compelling farmers to excessive antimicrobial use (AMU) for prophylaxis and/or treatment of diseases in order to reduce management costs and maximize returns on investment [2,10,12]. The sources of antimicrobial agents used by farmers vary widely but are mostly from hawkers and informal drug dealers who have little prescription knowledge [16,17], often leading to their misuse [12,18]. Over the counter sale of antimicrobial agents is widespread in low-income areas [19,20]. This situation is compounded by a lack of coordinated animal surveillance systems, weak enforcement of food safety regulations, lack of basic knowledge on AMU and resistance among the livestock keepers, and infection prevention and control (IPC) strategies in animal production [14,16,21,22].

In Tanzania, the drivers of AMU and antimicrobial resistance (AMR) in the animal and environmental sectors have been relatively less analyzed compared to public health [16,19,20,23]. The high level of AMU in a rapidly growing poultry and swine industry is likely to accelerate the development and spread of AMR beyond the animal and environmental compartments [17], with consequences to public health, animal production, and environmental contamination. The objective of this study was to assess the drivers of AMU and AMR including the knowledge, attitudes, and practices in poultry and domestic pig farming communities in the Msimbazi River basin, the most densely populated area in Tanzania. The basin serves as an important water source, and provides prime land for agriculture and animal grazing area, supplying most of the vegetables, fruits [24,25], poultry, eggs, and domestic pig products for the Dar es Salaam, the largest commercial city of the country that harbors six million people. The basin has very intensive agricultural and farming practices involving the use of manures, pesticides and antimicrobial agents, and is polluted with effluents and wastes from the largest pharmaceutical and commercial industries in Tanzania [25,26,27].

## 2. Results

A total of 113 farmers responded to the questionnaire, out of which 59 (52.2%) were females. Their age ranged between 18 and 69 years (mean age was 46.2 years and standard deviation of 11.8). The majority had secondary school education (46.9%, *n* = 53) and were married (63.7%, *n* = 72), and most of them (96.5%, *n* = 109) had been farming for more than six months (Table 1).

### 2.1. Knowledge and Practices Regarding AMU and AMR in Poultry and Pig Farming

About 88.5% of the respondents have knowledge of antimicrobials, and 69.9% indicate to know how to use them for the treatment of their animals. However, the majority (92%, *n* = 104) are not aware of AMR (Table 2). A significant relationship was detected between the respondent’s education level (X^2^ = 8.17, *p* = 0.04) and knowledge of antimicrobials. Farming experience has a significant relationship with the prudent use of antimicrobials (X^2^ = 6.88, *p* = 0.03) and the proper sourcing of the antimicrobials (X^2^ = 3.18, *p* = 0.04).

As shown in Table 3, 87.6% of the respondents indicated to have used antimicrobials, mostly following disease outbreaks. In most cases (95.6%, *n* = 108) diagnosis was based on clinical signs. The majority (84.1%, *n* = 95) had access to veterinary services, and most of them (89.4%, *n* = 101) bought antimicrobials from veterinary centers. The level of education was significantly associated with the frequency of using antimicrobials (X^2^ = 12.65, *p* = 0.04) and performing group treatment (X^2^ = 8.26, *p* = 0.04) irrespective of disease conditions. Stocking of antimicrobials was reported by many respondents (62.8%), mainly for 1–2 months. Group treatment of animals irrespective of disease condition was very common (88.5%, *n* = 100). There was a significant relationship between farming experience and the method used for disease diagnosis (X^2^ = 15, *p* = 0.04), access to veterinary services (X^2^ = 7.71, *p* = 0.01), storage of veterinary antimicrobials (X^2^ = 4.90, *p* =0.03) and group treatment (X^2^ = 16.42, *p* = 0.00).

### 2.2. Perception and Attitudes on Antimicrobial Use

Over half (59.3%, *n* = 67) of the respondents indicated farming must be accompanied by AMU, mostly for prophylaxis (97.3%, *n* = 110) rather than treatment (80.5%, *n* = 91) and the majority (66.4%) knew how to administer them (Table 4). The level of education was significantly related to the possibility of reducing antimicrobial use while attaining maximum production (X^2^ = 13.03, *p* = 0.01). Many (66.4%, *n* = 75) admitted that AMU in animal farming may pose a risk to human health, and some of them (53.1%, *n* = 60) agreed that it was possible to reduce antimicrobial use in animal farming and yet achieve maximum production. Farming experience has a significant relationship with the drugs not being effective (X^2^ = 6.97, *p* = 0.03) in treating animal diseases.

### 2.3. Perception and Attitudes on the Drivers of Antimicrobial Use and Resistance

About two-thirds of the respondents reported that the use of a combination of drugs is necessary for effective treatment, and nearly a half (48.7%) indicated using human medicines, and private drug sellers influence uncontrolled AMU. The majority of the respondents (92.9%, *n* = 105) acknowledged having inadequate knowledge on infection prevention and control of animal diseases, and most of them (95.6%, *n* = 108) indicated that veterinary and extension officers were inadequate. The use of human antimicrobials in animals (X^2^ = 8.37, *p* = 0.004), inadequate veterinary extension officers (X^2^ = 20.82, *p* = 0.000), and inadequate knowledge on infection prevention and control of animal diseases (X^2^ = 12.78, *p* = 0.005) were significantly associated with the level of education (Table 5). About two-thirds indicated that profit maximization necessitates the misuse of antimicrobials to shorten the period of farming, and 72.6% were aware of withdrawal periods.

### 2.4. Factors Associated with the Development and Spread of AMR in the Environment

Respondents indicated the following to be the main factors associated with the spread of AMR in the environment; disposal of solid wastes from the household (78.8%, 89), agricultural activities that involve use of animal manure (92.9%, *n* = 105), uncontrolled disposal of human and veterinary drugs (85%, *n* = 96) and use of river water for irrigation (92.9%, *n* = 105). As shown in Table 6, the farming experience was significantly related to the presence of agriculture activities that use animal manure (X^2^ = 11.62, *p* = 0.001); the use of river water for irrigation (X^2^ = 11.62, *p* = 0.001); and disposal of wastes from the household (X^2^ = 3.97, *p* = 0.004). Surprisingly, many (82.3%) did not think that pharmaceutical industries, which discharge effluents directly into the river, contributing to the spread of AMR.

### 2.5. Overarching Themes from in-Depth Interview and FGD

Analysis of qualitative findings from interviews and FGDs generated several themes regarding drivers for AMU and AMR. The main emerged themes were poultry and domestic pig diseases, access to antimicrobials, exposure to animal disease diagnosis and treatment, unavailability of government extension officers, waste management and environmental contamination, and the adherence to withdrawal periods.

### 2.6. Diseases Affecting Poultry and Domestic Pigs and Drugs Used for Treatment

**Figure 1:**
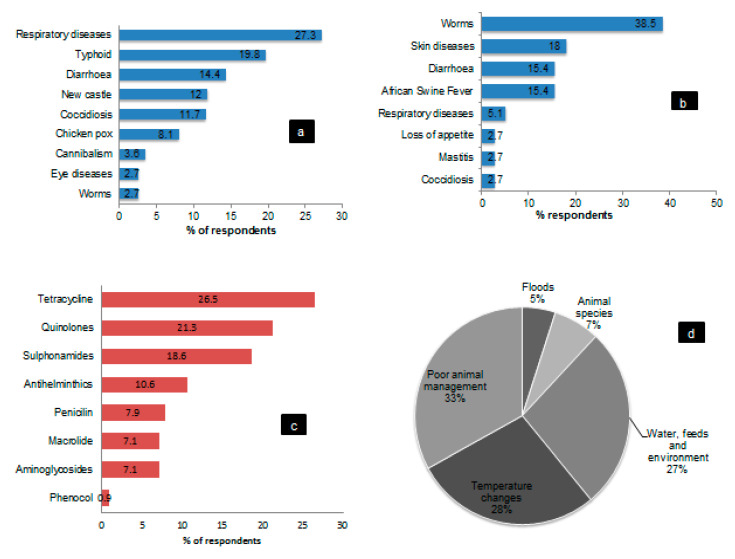
Respondent’s perception of the diseases affecting poultry and domestic pigs (**a**,**b**), classes of therapeutics used (**c**,**d**) the reason for a disease outbreak in the study area.

Interviewed farmers reported that the most common diseases encountered were typhoid, respiratory diseases, and diarrhea in poultry. Worms, skin diseases, diarrhea and African Swine Fever (ASF) were encountered in domestic pigs. Our study respondents mentioned different groups and names of the drugs used. Upon detailed analysis of the mentioned drug varieties, the research team grouped them into eleven classes with the main one being tetracycline and quinolones. The major causes for a disease outbreak that were mentioned included, poor animal management practices, temperature changes, contaminated water, feeds and the environment (untreated wastes, wastes from agricultural products and scavenging domestic animals (Figure 1).

### 2.7. Antimicrobial Use and Accessibility

The use of veterinary antimicrobials for treatment and prevention of diseases was familiar to all respondents. The findings from FGDs revealed that high usage of antimicrobials was attributed to the high frequency of disease occurrence and easy accessibility of antimicrobials from private veterinary drug sellers. It was reported that drug sellers were insisting and encouraging farmers to use drug combinations for effective treatment. Most of the FGDs participants reported that the disease burden was a result of the poor management systems. One of the FGDs participants reported “It is close to impossible to keep poultry or domestic pigs without using antimicrobials. Animals get sick frequently and the drugs are easily accessible. The sellers insist on the use of a variety of drugs to get the best production. Instead of helping us on appropriate use, they just want to sell more”. *Another FGDs participant had a similar comment stating*, “Drugs are used for treatment but mainly for prevention of diseases that frequently attack animals. The use of drugs save the life of the animals and consequently retain the investment capital”.

### 2.8. Experience in Diagnosis and Treatment of Diseases

Respondents admitted that the experience they had in animal farming has enabled them to be familiar with the diseases; therefore they were capable of detecting disease and treating their animals. They explained that they even collect samples from sick and dead animals for laboratory investigation. One of the focus group discussants had this to say: “The experience in animal farming enables one to tell the type of disease based on the signs and symptoms. The treatment instituted saves the lives of animals and the costs of consulting private extension officers. There is a possibility of either under/over-dosing because the amount of drug to be administered is guessed! Misdiagnosis is also likely to occur”. Furthermore, study respondents indicated that private veterinary/animal health practitioners were expensive and were mainly involved in the treatment of pig diseases such as mastitis, coccidiosis, and severe respiratory infections.

### 2.9. Availability and Accessibility of Government Extension Officers

Most of the respondents were not aware of the existence of the government veterinary services within their streets/wards. The private veterinary/animal health workers who owned most of the veterinary drug selling centers were working with the farmers. “It is like the government does not employ livestock extension officers anymore and if they do, they just stay in their officers probably because they don’t want to, or they lack facilities for visiting farmers” (key informant (KI) number 2). Another FGDs participant reported that “There is much trust in their services and it is likely that working closely with them will reduce diseases burden through improved animal management practices”.

### 2.10. Laws and Regulations Regarding the Handling, Use and Dispensing Veterinary Drugs

Almost all participants were not aware of the laws and regulations restricting veterinary drug handling, distribution, and sale. They reported that they obtain antimicrobials over the counter and have never experienced difficulties in obtaining drugs for their animals. On the other hand, veterinary drug sellers and animal feed manufacturers and sellers were aware of the presence of laws and regulations but acknowledged weak implementation. “There are laws and regulations but the implementation is weak” (KI number 7). However, none of them could name the specific laws and regulations.

### 2.11. Waste Management and Potential Sources of AMR Development and Spread in the Environment

The majority of the respondents admitted that the wastes generated from human sewage, animal waste and commercial factories wastes might contribute to AMR development and spread. They reported that solid wastes from domestic, human, and veterinary drug selling points were not sorted before collection and disposal. Additionally, wastes from the collection center were not disposed of on time or on a daily basis so they are most often scattered all over the place, thus they may become a potential source of AMR. “Waste from different households, effluents from the hospital including the nearby hospitals, industries like breweries and pharmaceuticals and others are lodged in this area during flooding. All these waste might contain residues of antimicrobials that contribute to the development of AMR in the environment” (KI number 5).

### 2.12. Withdraw Period of Antimicrobials

The use of human drugs was mentioned to be common in domestic pigs and poultry, mostly amoxicillin, doxycycline, ampicloxacillin, tetracycline, chloramphenicol eye drops, ciprofloxacin, metronidazole, and chloramphenicol. Almost all key informants and FGDs participants admitted to being aware of the withdrawal period, however, they found it difficult to implement for the fear of economic loss. One of the FGDs participants reported that “Farmers are aware of the withdrawal period but it is close to impossible to adhere with the time stipulated”. In another interview it was reported that “The capital invested is small, and it is a loan from the community serving cooperatives. The profit obtained from selling the poultry, domestic pig, and eggs are the ones used to run the family, feed the producing flock (for layers) and the remaining herd, and repay the loan. Waiting for the withdrawal period or discarding the eggs means extra costs which are difficult to overcome” (KI number 8).

## 3. Discussion

This study was conducted in Tanzanian’s most densely populated area with the highest concentration of both pharmaceutical and commercial industries, hospitals, pharmacies and drug sellers, intense use of pesticides and antimicrobials including insecticides in crop and animal farming, and intense environmental contamination [24,25,27,28,29]. Therefore, it is certainly the hottest hotspots for the spread of AMR organisms between humans, animals and the environment in the country. Additionally, the Msimbazi River drains to the Indian Ocean, which is international water therefore, the practice of AMU and AMR reflects wider implications.

We found a very high usage (87.6%) of veterinary antimicrobials in poultry and domestic pig farming mostly for prophylaxis rather than treatment. The high usage might be attributed to the prolonged exposure in animal farming coupled with negligence in adhering to good hygienic practices and other biosecurity measures. The higher proportion in this study corresponds to the one reported in Sudan, Ghana, Nigeria, and Cameroon where routine antimicrobials use are the normal practices even in absence of a disease outbreak [30,31,32,33]. Predominantly, farmers were making treatment decisions based on the presentation of clinical signs, which carries a high possibility of misdiagnosis, administration of inappropriate drugs and improper dosage. Similar studies in Sudan and Nigeria reported that antimicrobial use without involving veterinary practitioners and lack of laboratory findings in the diagnosis of animal diseases leads to improper use of antimicrobials [30,34].

Disturbingly, many farmers were using human medicines including amoxicillin, doxycycline, ampi-cloxacillin, tetracycline, chloramphenicol eye drops, ciprofloxacin, metronidazole and chloramphenicol to treat animals. Due to limited extension services and poor animal health delivery systems, farmers tend to buy veterinary and non-veterinary drugs from private drug shops and treat their livestock themselves. There is a high chance that farmers seek to maintain animal health welfare by using drugs that are cheaper, readily available, easy to use and more effective. The use of human medicine in animals proves the weakness in the implementation of laws and regulations governing handling, sale and use of antimicrobials both in humans and animals and limited extension services. This weakness spurs the problem of AMR organisms. This finding is consistent with [32,35] that antimicrobial use in food animals is accompanied by lack and or weak regulation, limited veterinary services and higher costs of private veterinary consultants.

Apparently, many farmers were stocking veterinary drugs at home, with a likelihood of poor handling and storage. The stocking of drugs might interfere with the active ingredient leading to reduced efficacy of the antimicrobials as reported by [36,37]. Moreover, metaphylaxis was very common, implying that even the healthy animals in a herd or flock were also treated thus escalating the chance of AMR development. Similar findings were reported in Tanzania [10] that stocking of veterinary antimicrobials was associated with improper usage leading to drug residues in animal products.

Frequently, farmers were using a combination of veterinary drugs on the advice of financially motivated veterinary drug sellers who do not have enough knowledge on drug use, side effects, proper dosage and withdrawal period taking advantage of inadequate veterinary services and extension officers. Similar findings were reported in Cameroon and Ethiopia that private veterinary services are expensive [33,38] and veterinary professionals are responsible for the abusive use of antimicrobials [39]. We found tetracycline and quinolones being the most used antibiotics due to being cheap, readily available and accessed easily, without restrictions [40], and are often sold by informal vendors at informal markets and even along the road [10,41]. Use of tetracycline in animals has been documented in Cameroon and Ghana probably since they are cheaper and have broad-spectrum activity against a variety of diseases [31,42]. The widespread use of quinolone observed in this study corresponds to the one reported in the previous studies [34,43,44] that quinolones are widely used in animals for diarrhea treatments and prophylactic despite the fact that they are expensive. Antibiotics are commonly sold during cattle auction days by informal vendors, such as petty traders and livestock keepers. Antibiotics found in markets like these are often unregistered, and therefore sold at very cheap prices (Abdu Hayghaimo- Former director of Veterinary services in Tanzania, personal communication, April 1, 2013). The quality of these medicines is undetermined.

We found many farmers to be aware of the withdrawal period, but most of them were not implementing it for several reasons including (i) economic loss (ii) regulatory bodies in Tanzania have not yet set withdrawal periods for veterinary drugs and farmers to rely, primarily, only on veterinary drug sellers. As a result, farmers rarely comply with the recommendations and the responsible regulatory authorities do also not monitor usage. Consequently, veterinary drug residues are likely to be present in food of animal origin, which poses a potential hazard to human health [45]. Failure to observe withdrawal periods in animal has been reported in Tanzania, Nigeria, Ethiopia, Sudan and Malaysia that it was among the reason for antimicrobial residues in food of animal origins and propagates development of antimicrobial resistance [10,15,36,46,47,48].

Additionally, our survey found different types and brands of antimicrobial used were from different sources that are not monitored and controlled. In Tanzania, the quality and quantity of veterinary antimicrobials are difficult to assess. This finding is comparable with the one reported by [30,49] that there is variation in the quality of veterinary antimicrobials which is in tandem with insufficient system for monitoring of antimicrobial use in animal production. While the Tanzania Food and Drug Authority (TFDA) performs quality assessments, on imported drugs at the port of entry, there is very weak post-market surveillance on veterinary medicines. We found the government does not control antibiotics included in animal feed, and that informal feed manufacturers found in small kiosks do not regulate the number of antibiotics included, leading to unnecessary exposure to antibiotics. During focus group discussions it became apparent that frequently drug importers, distributors and wholesalers supply drugs direct to consumers.

With regard to IPC, we observed poor housing with unhygienic conditions and limited air circulation in poultry houses. This most likely facilitated occurrence of most of the reported diseases by the farmers such as respiratory infections and typhoid in poultry and intestinal worms and skin diseases in domestic pigs, prompting increased use of antimicrobial agents. Poor housing conditions are contrary to the Animal welfare act 2008 [50] that requires animals to be kept in approved structures that conform to the quality hygienic and management practices. Collectively our findings suggest the acute need for the availability of livestock extension services (government or regulated private) at the local administrative level for the farmers to seek advice. We envisage that livestock extension personnel will be much trusted and their consultation service will be cost-effective and public health-relevant, as it will reduce the magnitude of AMU in animal farming and improve the safety of short-cycle stock derived food commodity. Furthermore, we recommend strengthening of the awareness and understanding of antimicrobial resistance by farmers and community level service providers (traders, extension personnel, and community-based animal health workers) through effective communication, education and training through collaboration between government and Civil Society organizations (CSO).

With regard to environmental contamination, respondents identified a number of activities taking place in the Msimbazi basin as potential drivers of AMR. These include effluents from households, hospitals, abattoirs and pharmaceutical and commercial industries, use of pesticides, flooding, and emptying of sewage into the environment during rainy seasons. Despite this, most of these activities are still on-going; posing health risks to humans and livestock by causing infections that are difficult to treat [25]. This is contrary to the Environmental Management Act, 2004 [51], which provides for a legal and institutional framework for sustainable management of the environment, including impact and risk assessments, prevention and control of pollution, waste management, environmental quality standards, public participation and compliance.

We found out that the existing Veterinary Acts (Code of Professional Conduct Regulations 2005, Livestock Policy 2006, Procedures for Registration Examination for veterinarian and veterinary specialists Regulations 2005, and Veterinary practice by Paraprofessionals Assistants Regulations 2005) [52,53,54,55], The hides, skins and Leather Trade Act No. 18 of 2008 [56], Veterinary Act No.16 2003 [57], Animal Welfare Act 2008 [50], and the Meat Industry Act 2006 [58] are weak and none of them specifically addresses issues of AMU and AMR in animals. Likewise, National Fisheries Policy of 2015 [59] aims at developing a sustainable, competitive, vibrant and more efficient commercialized fisheries and aquaculture industry that has no mention of AMU and AMR. We recommend that these acts and regulatory documents should be updated and work logistically to embraces a One Health (OH) approach, which is a cost-effective strategy for curbing AMR. The need for a cross-agency and cross-disciplinary collaborations has been suggested in some studies in [14,60] for the purpose of optimizing AMU, control quality, distribution, handling and awareness creation in human and animals and other related sectors.

On a positive note the government of Tanzania has developed a National Action Plan for Health Security 2017–2021 that aim to create and maintain active collaboration between the sectors for addressing health security using a “one health approach concept” so as to ensure that there is timely preparedness, and a consistent and coordinated response in the event of the occurrence of an event of public health concern. The plan is implemented under the guidance of the Prime Minister’s Office in order to achieve an Inter-ministerial Committee to administer the plan, and monitor and evaluate its implementation from all relevant line ministries. At the same time, the government has also developed a National action plan on antimicrobial resistance (2017–2022) [61], which adopted the One Health Approach, and has strategies that are related to monitoring and surveillance of AMR and antimicrobial consumption in human and animal, improving antibiotic stewardship and control the spread of AMR in both clinical and farm settings, and increase knowledge and public awareness on AMR and establish national governance for inter-sectoral actions.

Fortunately, there are a number of studies and OH AMU and AMR projects that are currently being implemented in Tanzania including i) supporting the National Action Plan on AMR in Tanzania (SNAP-AMR) that assesses prescribing practices in different health care settings as well as investigating community access and attitudes to antibiotics among household, community ‘drug’ shops and unregulated sources such as roadside traders and examine use in livestock by individuals with different levels of knowledge about AMR, e.g., district vets, community livestock officers and livestock holders; ii) the Fleming Fund Country Grant that aims to strengthen Tanzania’s national Antimicrobial Resistance (AMR) surveillance strategy by addressing the gaps in AMR data and strengthening antimicrobial stewardship. Ensuring that veterinary laboratories have access to a cadre of expert trainers tools, methods for analysis and interpretation and propose minimum data sets. Existing tools such as AfyaData can be adapted to collect AMR data and standardized protocols that will allow for the integration of data and comparative analysis among countries. These projects provide a platform for the implementation of the national action plan on addressing AMU and AMR.

## 4. Materials and Methods

### 4.1. Study Site and Design

**Figure 2:**
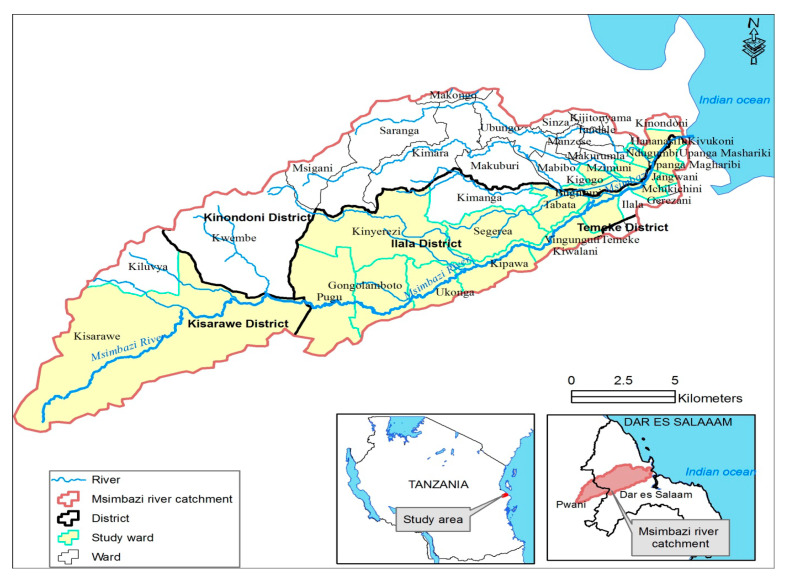
Map of the Msimbazi River catchment showing the study location. The study was carried out in 22 wards (indicated in yellow) located in Kisarawe, Ilala and Kinondoni districts. These included Pugu station, Gongolamboto, Ukonga, Kipawa, Segerea, Tabata, Vingunguti, Buguruni, Mnyamani, Ilala, Mchikichini, Jangwani, Upanga West, Kinyerezi, Tabata Liwiti, Kisarawe, Kazimzumbwi, Magomeni, Kigogo, Hananasif, Mburahati and Mzimuni. However, at the time of conducting the research, the administrative re-structure of the study area was not fully completed. Some of the wards are not seen, as they are the new ones where the geographical boundaries are still under preparation.

This was a cross-sectional study conducted between June and September 2019 in the Msimbazi River basin located in Kisarawe, Kinondoni, and Ilala Districts of Pwani and Dar es Salaam Regions in Eastern Tanzania. Msimbazi River, which is 45.25 km long, is the second-longest river within the Dar es Salaam region that originates from the higher areas of Kisarawe forest in the Pwani Region and discharges its water into the Indian Ocean (Figure 2). The basin, which covers an area of 271 km², is densely populated, harboring an urban population of about 1.2 million people. It is an important source of water for drinking, bathing, building, agriculture, and industries to the residents along the basin and its neighborhood. Multiple activities were undertaken, including crop and livestock farming, industrial commercial activities, fishing and sand mining. The basin supplies about 30% of the vegetables consumed in Dar es Salaam through the larger markets of Kariakoo, Ilala, Buguruni, Tazara, and Vetenari [27]. It is highly polluted by effluents originating from different sources, leakage of effluents from waste dumps, abattoirs, and domestic wastewater from septic tanks and pit latrines that are used by more than 70% of the Dar es Salaam population [25].

### 4.2. Sampling Strategy and Sample Size

Sampling and sample size for a survey: the sampling frame included all known poultry and domestic pig farmers. The sampling units were poultry and domestic pig farmers that were undertaking production at the time of the survey. Identification of the farmer to be interviewed was based on data provided by the ward livestock and/or agricultural officers and the respondents were randomly selected from the farmer’s list provided. The sample size was determined from the following formula *n* = z2p (1 − p)/e2 whereby z is the 95% confidence interval (which is 1.96), *p* is the estimated proportion of an attribute, and e is the standard error of the proportion (which is 0.05). The proportion of 90% reported by [12] was used to make a sample size of 138 respondents. However, in some of the wards, most potential farmers were vacated to allow for the construction of the Standard Gauge Railway project therefore the respondents consulted in this study were 113. The farmers selected were those with poultry and domestic pigs farmed for commercial purposes since they were likely to use antimicrobials to maintain animal welfare. Farmers who were veterinarians (either in practice or retired from service) were excluded from the study to avoid bias in the information to be collected.

Sampling and sample size for an in-depth interview and the focus group discussion (FGDs)*:* participants for an in-depth interview and the focus group discussion were purposively chosen with the aid of the extension officers at the ward level. They were recruited through mobile calls and face-to-face conversations. Five FGDs consisting of 8–12 individuals and 8 in-depth interviews were conducted. The number of respondents consulted in this study was based on the guiding principle of data collection to the point of saturation [62]. The qualitative data collection was stopped at 8th interview and 5th FGDs after the research team satisfied that there was no more new or relevant data regarding the emerging themes [63].

### 4.3. Data Collection

Face-to-face interviews: pre-tested questionnaires were designed in English and the contents were translated into Kiswahili during administration. The questionnaire (supplementary information 1) was digitalized into AfyaData, a mobile digital data application [64] that was installed into a smartphone. Interviews were conducted at the household/farming premises. The heads of the households were the main respondents. In the absence of the head of households, information was sought from any other adult (>18 years old) occupants who were engaged with livestock keeping. The questionnaire contained close and open-ended questions covering four main themes: (i) socio-demographic information such as sex, age, marital status, education level, and experience in animal farming; (ii) knowledge, attitude, practices and perceptions on AMU and AMR in poultry and/or domestic pig farming; (iii) drivers of AMU and development of AMR in animal farming; and (iv) environmental contamination resulting from animal wastes. Additionally, observation of the antimicrobial used and stored, type and hygiene of the housing and personnel, type of feeds, waste management, and disposal were done and recorded into a different sheet. The closed-ended questions were coded into categorical variables and the open-end questions and observed information was organized into subcategories before analysis.

Focus group discussions and in-depth interviews: the FGDs and in-depth interview participants were both male and female selected from farmers, poultry and domestic pig buyers, and community members. The aim was to collect complementary information from different categories of respondents who are directly or indirectly involved in the AMU and are at risk of exposure to AMR. The time taken for each FGD was 2–3 h and the in-depth interview was 30–45 min. The FGDs and interview guide questions (Supplimentary information 2 and 3) were prepared to capture their views about drivers of AMU and AMR, availability and accessibility of veterinary drugs, animal treatment practices, environmental contamination, waste management practices, and the role of the government concerning policy, regulation, and control of veterinary antimicrobials.

### 4.4. Data Management and Analysis

The collected quantitative information was sent to a server, integrated and mapped to produce information that was transferred to the Microsoft Excel spreadsheet (supplementary information 4) and analyzed using the SPSS version 20.0 for Windows (IBM Corp., Armonk, NY, USA) software. The outcomes concerning the drivers that were assessed using the 4-point Likert scale were initially described with numbers and percentages. They were then dichotomized as “True” versus “False” such that the value of “strongly agree” and “agree” (as True) versus “Strongly disagree” and “disagree” (as False). Descriptive statistics such as frequency and percentages for categorical variables were determined to generate and summarize the results in tables and figures. Mean, median and the standard deviation were computed for continuous variables. The Chi-squared test was conducted to identify the association between the outcome and explanatory variables. The value of *p* < 0.05 was considered significant. Information from the focus group discussion and the interview (supplementary information 5) was subjected to transcription, creating categories, and then coded into categorical variables as per (30) and were analyzed manually. The results for the overarching themes were presented.

### 4.5. Ethical Considerations

The Medical Research Coordinating Committee of the National Institute approved this study for Medical Research of Tanzania (Reference No. NIMR/HQ/R.8a/Vol. IX/3133) and Muhimbili University of Health and Allied Sciences (Permit No. DA.282/298/01.C). Written consent form (supplementary information 6) was provided to the participants to sign before commencement of the interview. Participants were informed of the opportunity to withdraw from the study at any time without prejudice.

## 5. Conclusions

This study found a high usage of veterinary antimicrobials primarily for prophylactic purposes among poultry and domestic pig farming communities. Most farmers have inadequate knowledge of IPC and antimicrobial use and have limited access to veterinary and extension services, paving the way for self-treatment and opportunism by profit-driven non-professional veterinary drug sellers. The existing veterinary legal framework is weak and is hardly implemented due to a number of reasons including inadequate veterinary and extension services. Our study proposes the implementation of OH interventions that focus on optimizing antimicrobial use in animals and humans and measures to minimize environmental contamination to minimize the occurrence of infections and use of antibiotics and promote health and productivity to realize the sustainable development goals (SDGs). Additionally, the study calls for more research to evaluate the active ingredients of antimicrobials consumed in specific animal species in the study area and across the country in order to get the true reflection of the magnitude of AMU in food animals.

## Figures and Tables

**Table 1:** Socio-demographic characteristics of participants for questionnaire interviews.

Characteristics	Number (*n* = 113)	Percentage (%)
**Sex**		
Male	54	47.8
Female	59	52.2
Marital status		
Married	72	63.7
Single	28	24.8
Widower/widow	13	11.5
Age (years)		
18–34 years	18	15.9
35–44 years	46	40.7
45 years and above	49	43.4
Education level		
None	4	3.50
Primary school	38	33.6
Secondary school	53	46.9
College education	18	15.9
Main occupation		
Pig farming	4	3.50
Poultry farming	25	22.1
Pig and poultry farming	19	16.8
Pig and poultry farming with other business	65	57.5
Experience in animal farming		
Less than 6 months	4	3.50
More than 6 months	109	96.5

**Table 2:** Number and percentage of respondents on the knowledge of antimicrobial use (*n* = 113).

Characteristics	n (%)	Ever Heard of Antimicrobials (Yes)		Knowledge on Antimicrobial Use in Animals (Yes)		Source of Knowledge on Antimicrobial Use (Family Members, Friends and Neighbours)		Adequate Knowledge on Management of Farm Animals (Yes)		Knowledge on Antimicrobial Resistance (No)	
		Χ^2^	*p*-Value	Χ^2^	*p*-Value	Χ^2^	*p*-Value	Χ^2^	*p*-Value	Χ^2^	*p*-Value
Sex		0.22	0.64	1.28	0.26	3.31	0.18	1.51	0.22	2.04	0.15
Male	54(47.8)										
Female	59(52.2)										
Age		0.05	0.98	0.87	0.93	0.96	0.62	6.46	0.09	0.31	0.86
18–34 years	18(15.9)										
35–44 years	46(40.7)										
45 years and above	49(43.4)										
Education level		8.17	0.04 *	4.56	0.60	12.07	0.01 *	0.18	0.91	6.47	0.09
None	4(3.5)										
Primary school	38(33.6)										
Secondary school	53(46.9)										
College education	18(15.9)										
Experience in farming		16.42	0.00 *	6.88	0.03 *	3.78	0.04 *	0.68	0.41	2.84	0.92
Less than 6 months	4(3.5)										
6 months and above	109(96.5)										

* Significant *p* value < 0.05; Χ^2^ = Chi square test.

**Table 3:** Number and percentage of respondent’s response to the practice related to antimicrobial use.

Variables with Respective Response		Sex		Age		Education Level		Experience in Farming	
	N (%)	Χ^2^	*p*-Value	Χ^2^	*p*-Value	Χ^2^	*p*-Value	Χ^2^	*p*-Value
Ever used antimicrobials (Yes)	99(87.6)	1.74	0.18	2.95	0.62	9.46	0.02 *	5.40	0.02 *
Frequency of using antimicrobials in animals (Following disease outbreak)	63(55.8)	3.07	0.22	5.84	0.21	12.65	0.04 *	1.32	0.85
Methods used in diagnosis of the diseases affecting domestic pigs/poultry (Clinical signs)	108(95.6)	3.31	0.58	2.56	0.28	2.13	0.55	4.15	0.04 *
Provider of treatment to animals in your farm (Farmer/ family member)	85(75.2)	2.49	0.11	1.96	0.38	7.14	0.68	1.42	0.23
Time of treatment in domestic pigs and poultry (During disease occurrence)	70(61.9)	1.72	0.42	4.54	0.34	15.42	0.02 *	6.59	0.37
Access to veterinary services (Yes)	95(84.1)	7.71	0.05 *	3.06	0.22	4.96	0.18	10.81	0.01 *
Source of drugs (Veterinary centres)	101(89.4)	2.20	0.65	2.07	0.35	1.51	0.92	1.49	0.48
Possibility of underestimating dose during disease treatment (True)	71(62.8)	3.01	0.97	1.33	0.52	1.21	0.75	2.26	0.61
Stocking of antimicrobials at home (Yes)	71(62.8)	2.57	0.45	3.90	0.14	1.37	0.71	2.54	0.11
Time for drug storage (1-2 months)	58(51.3)	2.56	0.1	1.88	0.39	1.88	0.59	4.90	0.03 *
Practicing group/mass treatment in domestic pig/ poultry farming (True)	100(88.5)	2.21	0.64	2.19	0.91	8.26	0.04 *	16.42	0.00 *

* Significant *p* value < 0.05; Χ^2^ = Chi square test.

**Table 4:** Respondent’s perception and attitudes on the use of antimicrobials.

Variables with Respective Response	N (%)	Sex		Age		Education Level		Experience in Farming	
		Χ^2^	*p*-Value	Χ^2^	*p*-Value	Χ^2^	*p*-Value	Χ^2^	*p*-Value
Farming of poultry/domestic pig must be accompanied by antimicrobial use (True)	67(59.3)	1.32	0.25	2.43	0.29	3.92	0.27	2.20	0.16
Farmers who use antimicrobial are very knowledgeable on how to administer them (False)	71(62.8)	2.01	0.98	6.56	0.04 *	3.40	0.93	2.45	0.12
Antimicrobial usage in domestic pigs/poultry farming may be risky to human health (True)	75(66.4)	0.54	0.46	1.18	0.55	6.73	0.08	3.14	0.71
Antimicrobials are used to prevent diseases in poultry/domestic pigs (True)	110(97.3)	2.82	0.09	4.49	0.11	3.86	0.83	3.11	0.74
Animal deaths are highly reduced through the use of antimicrobials (True)	88(77.9)	1.92	0.17	1.72	0.42	2.12	0.55	1.87	0.17
It is possible to reduce antimicrobial use in animal farming and yet achieve maximum production (True)	60(53.1)	1.15	0.9	1.57	0.75	13.03	0.01 *	2.02	0.89
Some drugs are not effective to treat particular infection(s) (True)	93(82.3)	2.05	0.83	3.46	0.79	5.87	0.12	6.97	0.03 *
Seasons when experiencing most disease occurrence (Rainy season)	49(43.4)	2.53	0.77	2.72	0.77	5.81	0.45	3.57	0.75

* Significant *p* value < 0.05; Χ^2^ = Chi square test.

**Table 5:** Respondent’s perception and attitudes on the drivers of antimicrobial use and resistance in animal farming.

Variables with Respective Response	N (%)	Sex		Age		Education Level		Experience in Farming	
		Χ^2^	*p*-Value	Χ^2^	*p*-Value	Χ^2^	*p*-Value	Χ^2^	*p*-Value
Use of combination of drugs is necessary for effective treatment of animal diseases (True)	73(64.6)	7.35	0.007 *	0.53	0.85	0.57	0.75	0.19	0.66
Antimicrobials used in humans are also used domestic pig and poultry farming (True)	55(48.7)	7.53	0.006 *	6.16	0.04 *	8.37	0.004 *	1.15	0.28
Private veterinary drug sellers leads to uncontrolled handling and use of veterinary drugs (True)	81(71.7)	2.40	0.12	0.43	0.81	1.88	0.59	0.96	0.33
Antimicrobials are used to enhance growth of poultry and/or domestic pig (True)	67(59.3)	3.01	0.99	0.17	0.92	3.91	0.27	0.42	0.52
Inadequate veterinary/extension services contributes to drugs administration by farmers (True)	108 (95.6)	0.13	0.72	0.92	0.63	20.82	0.000 *	4.15	0.04 *
It is better to have a stock of veterinary drugs at home (True)	56(49.6)	0.88	0.53	1.06	0.58	1.01	0.79	4.07	0.04 *
Inadequate of knowledge on infection, prevention, and control of animal diseases (True)	105(92.9)	1.02	0.89	4.53	0.10	12.78	0.005 *	2.02	0.16
Profit maximization necessitate misuse of antimicrobials to shorten period of poultry/domestic pig farming (True)	69 (61.1)	2.42	0.12	0.72	0.7	16.15	0.001 *	1.21	0.64
Awareness of withdrawal periods among poultry/ domestic pig farmers (True)	82(72.6)	3.12	0.07	3.39	0.18	0.38	0.94	1.06	0.3

* Significant *p* value < 0.05; Χ^2^ = Chi square test.

**Table 6:** Respondent’s perception of the factors associated with antimicrobial resistance spread in environment.

Variables with Respective Response	N (%)	Sex		Age		Education Level		Experience in Farming	
		Χ^2^	*p*-Value	Χ^2^	*p*-Value	Χ^2^	*p*-Value	Χ^2^	*p*-Value
Solid wastes containing drug left over from households are disposed directly into the environment (Yes)	89(78.8)	2.64	0.1	4.78	0.92	5.32	0.14	0.35	0.85
Agricultural activities that use manure obtained from animals (Yes)	105(92.9)	0.75	0.38	1.29	0.52	12.78	0.005	11.62	0.001 *
Household slurry released directly into rivers (Yes)	45(39.8)	6.25	0.01 *	1.01	0.61	6.37	0.95	0.18	0.67
Leakage of surface water pipes that provide mixing of manure and other wastes into water bodies (Yes)	68(60.2)	0.33	0.56	2.39	0.82	0.36	0.85	0.38	0.54
River water used for irrigation and farming activities (Yes)	105(92.9)	2.01	0.89	3.17	0.92	3.38	0.34	11.61	0.001 *
Pharmaceutical industries discharge effluents directly into the river (Yes)	20(17.7)	0.57	0.48	1.12	0.57	3.91	0.27	0.15	0.69
Frequent floods during rainy season (Yes)	78(69)	1.32	0.25	4.61	0.1	1.52	0.67	0.34	0.56
Uncontrolled disposal of human and veterinary drugs from different sources (Yes)	96(85)	2.29	0.13	0.64	0.79	4.45	0.22	3.96	0.04 *
Access to emptying sewage systems during flooding (Yes)	86(76.1)	0.24	0.62	3.89	0.14	1.57	0.67	3.03	0.96
Disposal of wastes from the household into rivers (Yes)	89(78.8)	1.01	0.92	6.98	0.03 *	0.22	0.57	3.97	0.04 *

* Significant *p* value < 0.05; Χ^2^ = Chi square test.

## Supplementary Materials

The following are available online at https://www.mdpi.com/2079-6382/9/12/838/s1, The information presented are the questionnaire used for the survey (Supplimentary 1); the FGDs and in-depth interview guiding questions (supplementary 2 and 3 respectively) used to generate information for the study is attached. Others are the Excel sheet used for analysis (supplementary 4); information from the key informat (supplementary 5) and the consent form (supplementary 6).
