# Supplementary material for: Assessment of Drivers of Antimicrobial Use and Resistance in Poultry and Domestic Pig Farming in the Msimbazi River Basin in Tanzania
Source: Antibiotics (Basel). 2020 Nov 24;9(12):838. doi: 10.3390/antibiotics9120838 (PMC7760815; doi:10.3390/antibiotics9120838)
Supplement: Supplementary file 1 [file antibiotics-09-00838-s001.pdf]

Responses from the Focus Group Discussions
Location: Tabata Segerea Ward
Interviewees: Livestock keepers (men and women)

Five women and three men aged between 28–59 years were interviewed in Tabata Segerea ward. All of them have been living in Segerea for more than three years and they are owner of the land. Their income comes from poultry and pigs farming together with other business. The mode of farming is mainly intensive and commercial because they are farmed for commercial purposes and the area involved is small enough to practise extensive. All respondents have been involved in farming of short cycled animals for more than two years except one who has farmed for more than six months. The number of poultry kept ranged from 300 – 3000 and for the pigs were 10 – 70 animals. The challenge with large animal farming is that the municipal by-laws insist on keeping not more than 4 animals so they are forced to confine and manage them in strictly intensive mode of production. This farming system is good for protecting pigs from the community member that do not want to hear about pigs and pig products.

According to the group, the use of antimicrobial agents in pigs and poultry is high especially in poultry farming. The birds are frequently attacked by diseases such as coryza, typhoid, coccidiosis, respiratory diseases and diarrhoea. The use antimicrobial is to avoid economic loss arising from animal death. In pigs the frequently occurring diseases are worms, respiratory diseases, skin diseases, mastitis, iron deficiency and diarrhoea. The farmers themselves are experienced enough to do diagnosis through clinical signs and symptoms. In rare cases especially when they suspect of massive death of the animal they send sample of the dead or sick birds to the veterinary centres for diagnosis. Some of these centres do have laboratory where they open the birds or carcases to know the type of diseases. When they fail at the veterinary centres then the diagnosis have to be made from the central veterinary laboratory – head office. They once sent the samples of animals and feeds for analysis. As with pigs the veterinary doctor are consulted to do the treatment. In case he/she fails and the condition of the animal is deteriorating, the animals are slaughtered to avoid losses.

Veterinary drugs are available with no restriction but most of them especially those made from Tanzania are not effective. In this case farmers have to opt for combination of drugs or use particular drug for more than three times to ensure effective treatment. Even those injectable ones such as penicillin are also not effective. There is high usage of human drugs such as Amoxycline, Doxycline, Ampiclox, Tetracycline, Diazepam (Valium), Chloramphenicol eye drops and Chlormpheniramine (Piriton) are the best option. They are readily available and cheap compared to the animal drugs. As with Valium and Piriton they put 10-12 tablets in 20litres of water and they are effective in treating Coryza. The uses of local herbs are also commonly practiced and they are Aloevera, Neem leaves in poultry and used grease in pigs. The commonly veterinary drugs used are Penicilin, ivomec, piperazine, OTC 20% and 50% and pig booster in pigs. In poultry the drugs used are Limoxin (Chlorohydrate OTC), Amprolium, Neomycine, Tylodox (Tylosine and Doxycline), Ganadexil (Enrofloxacilin), Enrovet (Enrofloxacilin), Sulphadiazone trimethoprim, Trimapharm, Oxytetracycline, multivitamin and pig booster, broiler booster and egg booster.

The frequency of diseases is mainly during the rainy season in pigs but in poultry it is throughout the year. Sometimes the causes of diseases are the feeds and the animal batch although the management and hygienic practises has a role to play. Due to infective treatment of most of the drugs, poultry are sometimes injected with Penicillin. The drugs are infective actually they are fake drugs especially those made from Tanzania. Some have stopped buying drugs that are made within the country but rather prefer the imported ones although they are very expensive.

According to the group the role of extension officer is of great importance. They rarely visit farmers. They wish that they could be available throughout to advise them from the initial stage of construction of the pigs and poultry houses. If possible the government has to plan on how to distribute free antimicrobials to farmers as it was the case during Nyerere's era. In some cases the few available extension officers lack facilities for disease diagnosis and transport medium. They have never heard about the laws governing handling and sale of veterinary drugs and if they are there then they are not implemented effectively.

Regarding management practices, the excessive use of antimicrobials is not good. They have not heard about antimicrobial resistance but treating animals more than three times using different drugs imply that the organisms are resistant to the drugs. Even the use of human drugs to treat animals is because the veterinary drugs used have become resistant. The seller has an influence towards uncontrolled use because they are the one insisting on the use of variety of drugs. They are aware of the withdrawal period but it is difficult to implement for the fear of economic loss. Even the eggs are sold while the poultry's are under treatment.

Regarding waste management, the wastes are not sorted to isolate veterinary and/or human drugs. Every individual house and business centre including veterinary and human drug stores collects the wastes and put in a bag or container waiting for the municipal vehicles to collect. In most cases wastes are not picked on time so they are scattered alongside the street. This provides rooms for the expired and unused drugs to be mixed with other wastes and contribute to occurrence of AMR problem. Water pipes are leaking and there is close contact between the water and other wastes including drugs left over. Manure from the livestock kept in this ward is used by the vegetable growers along the valley. It is a highly valuable product sold up to 5000/= per bag of 50kg.

Regarding flooding, it is a common problem during rainy season. Last year some of the farmers lost their animals from flooding. It is during this time that community along the river empty their latrines. Diseases such as cholera are common during this time.

Difficulties

- Lack of vaccines such as Newcastle
- Presence of fake drugs especially those made within the country
- Laboratory to refuse to issue certificate of the diagnosis of the problem identified despite the fact that they have paid for the service
- Loss of large number of poultries from the reason explained as due to type of feed used. Farmers lost up to 1000 chicken while at the time of selling.

Location: Ukonga ward
Interviewees: Livestock keepers, veterinary drugs sellers and animal feed manufacturers

A group of men and women with different economical activities were interviewed. They were poultry and pig keepers, veterinary drug sellers and buyers of pigs and poultry along the area. All interviewee have been doing their respective activities for more than three years. They are owner of the land in Ukonga ward and lived for more than five years.

Regarding animal farming: Intensive farming is commonly practiced in commercial mode of production. There is some few extensive farming of local chickens. This kind is important to utilize as much as possible the small area of land to raise large number of animals. As with pigs apart from being a means of utilizing land effectively, it also aid in protection of the animals against the community members who thinks that these animals are not supposed to be domesticated. Also the municipal by-laws require farmers not to have more than four large animals; therefore it is also a means of protecting them to be seen by the neighbours and municipal workers.

Diseases affecting animals: These differ between poultry and pigs although with other large animals such as dairy cattle, some of them do resemble. Pigs are mostly affected by worm, iron deficiency, respiratory diseases, mastitis, diarrhoea, ASF, skin diseases and coccidiosis. With poultry the disturbing diseases are typhoid, Coryza, Respiratory diseases, Coccidiosis, New castle, fowl pox, eye diseases and cannibalism. The diseases occur throughout especially in poultry but they are worse during rainy season. This is likely because of the nature of the houses which do not allow proper air circulation, allows contaminated water during rainy season and are wet in most of the times.

Disease diagnosis and treatment: The respondents are experienced enough to make diagnosis and treatment of their animals. In severe cases where treatment fails, they send the live or dead animals to the veterinary centre of the central veterinary laboratory of the Ministry of Livestock and Fisheries for diagnosis. The knowledge they have was enriched by attending seminars provided by some of the animal feed manufactures and veterinary drug producers. With pigs, the private veterinary or animal health providers are consulted to come and make treatment at the site.

Antimicrobial use and accessibility: Drugs are available and easily accessed from the veterinary centres. It is a matter of which kind of drugs the farmers prefer. There are local produced and imported drugs. Antimicrobials are highly used in the pigs and poultry production. It is close to impossible to farm these animals without being using antimicrobials. The animals themselves get attacked to varieties of diseases some of them have become resistant but also even the nature of the housing predispose occurrence of diseases. Due to small investment capital it is difficult to have modern houses that will protect or reduce to the larger extent occurrence of diseases.

Drugs used to treat pigs and poultry: Several drugs are used in pigs and poultry. With poultry they are Tetracolivit (tetracycline), Oxytetracycline, Neoxychick (Neomycin), Amprolium, Ganadex (Enrofloxacilin), Tylodox (Tylosine and Doxycline), Fluban (Enrofloxacilin), ESB3 30% (Sulfaclozine Sodium), Interim (Sulfadiazine Trimethoprim), Trimafarm 30%, Enrovet (Enrofloxacilin), multivitamin, pig booster, broiler booster, and eggs booster. In pigs the drugs used are Dextran 20% (Iron), Ivermectin, Multivitamin, Sulfardimidine, Limoxin (Chlorohydrate OTC), Oxytetracycline 20% and Oxytetracycline 10%. Combination of drugs is practiced to ensure effective treatment because using less than one drug in most cases the animals are not get treated. The animals are sold while under treatment although they are aware of the withdrawal period. It is difficult to implement since waiting for four or seven days recommended means a huge loss that they cannot afford. Some of their fellow's claims to follow the regulation but in actual sense it is not true. The poultry buyers explained that they have been buying from farmers and selling sick animals they are not staying for a long time with the poultry. This means that all the drug residues that the animal used are freshly consumed at the end products.

Work with extension officers, laws and regulations: About half of the respondents are not aware of the existence of livestock/veterinary officers at the ward level. They work with the private veterinary and animal health workers who are known to own veterinary drug stores. They get advice according to the problem that what brought them to the centres. There are some issues that demand site visit for proper advice and they would wish to be visited. Except for veterinary drugs sellers the rest of the interviewee were not aware of the laws and regulation that govern sale and handling of veterinary drugs. However, the veterinary drug sellers admit that implementation is not that much strict, what they are keen enough is to pay licence and taxes as per authority's requirements. Awareness on the danger of AMU and the problem of AMR needs to be done by the extension officers while visiting farmers but also there is a need to have special programs on Radio and TV.

Waste management and disposal: Wastes generated at household, pharmacies, business centres and other areas are collected by the municipal. Every producers of the waste are required to put them in bags/containers so that when the municipal vehicles pass they just collect them. There is likelihood that even expired drugs from the human and veterinary centres are mixed with other form of wastes thus exposing the community to AMR problem. Manure coming from the animal farming is used in agricultural activities within the ward especially along the valley where the main activity is vegetable growing during dry season.

Flooding and AMR occurrence: Flooding is a major problem during rainy season. Some of the houses that were very close to the river are flooded and the owner used to vacate for some time. The communities and industrial along the river do empty their effluent to be washed away by the water. With that there might be a possibility of spreading of resistant organisms from one point to another.

Location: Jangwani ward
Interviewees: Community members, Veterinary drugs sellers and Livestock keepers
Twelve participants were involved in the interview. Seven were owners of the land and five were tenants. The group comprised members from Mzimuni and Jangwani wards. The land owner has stayed for more than 15 years in the area. Two of the tenants have lived for three years, one for two tears and the other two for five years.

Animal farming: Farming of animals in the area is conducted under difficult environment since the land owner's thinks it is more profitable to have houses for renting rather than leaving space for animal keeping. Few people conduct intensive farming of poultry and very few do keep pigs because the areas are surrounded by Muslims who do not like pigs. It is very difficult to protect the pigs as sometimes they find means of killing them. Another problem that made farming activities to be difficult is the problem of flooding. Most farmers have incurred frequent loss during rainy season as the animals are taken by floods. However the business is very profitable because the products are obtained close to the potential markets and it is a very profitable.

Antimicrobial use and accessibility: As the area seen, farming of pigs and poultry is nothing without the use of antimicrobials. There is high rate of diseases which necessitate use of antimicrobials throughout the production. The antimicrobial agents are easily accessible in shops located in Magomeni and Kariakoo market. These are the main suppliers of both local and imported drugs.

Animal diseases and frequency of occurrence: The environment is almost highly compacted and contaminated. The houses where animals are kept are of poor quality therefore animals are frequently attacked by diseases. The situation is more critical during rainy season. Poultry suffers from Coryza, Respiratory diseases, Diarrhoea, Typhoid, New castle (for those not vaccinated), fowl pox and Coccidiosis. As with pigs the common diseases are worms, diarrhoea, ASF (these do occur seasonally), fever, respiratory diseases, mastitis, and skin diseases. They think that the type of feeds given to pigs (mostly food left over from the restaurants) is the cause of diseases especially worms.

Diseases diagnosis and treatment: All interviewee have been in animal farming for more than two years therefore they are knowledgeable enough to identify sign and symptom of many diseases affecting their animals. Farmers do conduct treatment of both pigs and poultry. In some rare instances they have to call private veterinary or animal health attendants from the veterinary drug sellers to come for treatment.

Drugs used to treat pigs and poultries: Varieties of drugs are used. These depend on the type of clinical signs presented but also advise from the seller. Sometimes the seller had an influence on the type of drugs. In most cases they insist on combination of drugs probably because they want to increase the sale. Drugs such as ESB3, Ganadex, Enrovet, Fluban, Chloramphenicol, Tylodox, Trimazine, Limoxin, Neoxychick, Amprolium, Multivitamins,

Broiler booster, pig booster and egg booster. In pigs they use pig booster, multivitamin, iron injection, OTC 20% and 10%, Limoxin (Chlorotetracycline), Piperazine, Ivermectin, Sulphur, Penstrep, and Tylosin. The problem with these drugs is that apart from being expensive, they differ in effectiveness. Some works well while others are not. In this case the use of human drugs is sometimes become important because they are effective and cheap. The human drugs used are Piriton, Amoxycline, Doxcyciline, Tetracycline, Ampiclox, Ciprofloxacin, Metronidazole, and Chloramphenicol eye drops.

Work with extension officers: There is deficit of veterinary and or livestock officers in most of the ward especially those located close to the city centre. The available ones work in more than three wards so it is difficult to visit the farmers. Additionally, they assume that there is almost no commercial farming in these areas. They work with private extension officers especially those that own veterinary centres and others introduced to them by their relatives. They are not sure whether there are government regulations on veterinary drugs since they can easily access them from the seller. The awareness on AMR is highly needed as majority of the community members are not aware. They know that some drugs for both human and animals are not effective or they are of substandard quality. They think the government needs to do much on awareness. According to their surrounding they believe that animal farming without the use of antimicrobials is very difficult. They are aware that the natures of the animal houses are of poor quality so even if they got advise on how to improve, it will be difficult for them as the construction will either require more areas or be in a form of raised platform which is expensive.

Waste disposal and management: Jangwani area is like a waste disposal point. All the wastes from different sources are clogged in Jangwani. When the river floods the wastes enter peoples household. Considering the fact that these wastes comes from different areas including household slurry, garages, industries along Nyerere road, Vingunguti abattoir, Tanzania Breweries and the Muhimbili National hospital, they consider that the areas are the potential sources for the problem. There are some houses where water has been clogged since last rainy season to date while in the nearby houses, routine activities are running smoothly. The disposed wastes are not sorted to separate the hazardous from the non-hazardous. Although community are required to collect wastes and put in a collection centres for the municipal vehicle to pick, those close to the valley do not do so. They throw their wastes to the river. There is no clear road for the vehicles to pass through therefore these community take advantage by throwing wastes directly to the river.

Problem of flooding: This is the most common problem in the areas. Every year the situation is getting worse and people die due to floods. They think that flooding contributes to AMR because wastes from different sources are mixed together.

Location: Mivinjeni Street Office
Interviewees: Livestock keepers, pigs and poultry buyers and other community members
A group of eight people were interviewed. They have been living in this area for more than three years. Five are land owners and three are tenants.

Animal farming and mode of production: Poultry is the most type of farming existing although few of them keep pigs. The mode of farming is intensive due to small areas and the municipal by-laws that restrict the number of animals. Both poultry and pigs has a huge potential market ranging from the surrounding community members, the Buguruni market and the formally pig market known as "Mabanda ya nguruwe ya Sukita".

Antimicrobial use and accessibility: They insist that the use of antimicrobials in animal farming is high. This is due to disease frequency which keeps on attacking their animals throughout the year. There is no restriction on assessing both veterinary and human antimicrobials.

Diseases affecting animals and the frequency of occurrence: Several diseases affect poultry and pigs. Poultry are affected by Typhoid, Coryza, Coccidiosis, Respiratory diseases, Cannibalism, Newcastle (when the poultries are not vaccinated as required), and Diarrhoea. Pigs are affected by worms, iron deficiency, diarrhoea, ASF, fever, skin diseases and respiratory diseases. Frequency of diseases is throughout although it is severe during rainy season. During this time poultry get severe coryza, reduced eggs production and stunted growth. The pig suffers from worms and diarrhoea.

Diagnosis and treatment of sick animals: Clinical signs are the most common way used to know if the animal is sick and once suspected treatment is instituted by the farmers themselves. They are lucky that they are close to among the largest veterinary centres in Tanzania, the Farmbase where if treatment fails, they took the sample of live and/or dead animals for examination. With pigs they call a veterinary doctor from the company to do the further treatment including sample collection of blood and/or fecal materials. The service is expensive but what matters is for the animals to be cured.

Drugs used: In poultry they use Tetracycline, Oxytetracycline, Enrovet, Ganadex, Fluban, Neomycin, Tylodox, Sulphadimidine, Trimazine, Ampicilline, Multivitamin, Chloramphenicol, and Amproluim. To enhance growth and eggs production they use Pig booster, broiler booster and egg booster. In pigs, the drugs used are Ivermectin, Penstrep, Iron injection, and sulphur. Also pig booster is mixed with feed to enhance growth. The human and local drugs used are Aloe Vera leaves, tetracycline, ampicilline, ciprofloxacin, ampiclox, piriton, Valium, and Vitamin B complex. Also grease and dirty oils is used to treat skin diseases in pigs.

Work with extension officers: Very few farmers (only 3) are aware that there are livestock officers at the ward level. They normal work with the private owned officials and not the government. However, these private ones do not visit them, rather they are waiting for the

farmers to go and/or call when they need the service. They would wish to work close with them since they have much trust on government officials rather than private who are money oriented people. Little is known about the laws and regulation on veterinary drugs handling and distribution. They have seen people from the TRA and those responsible for licensing visiting and inspecting the veterinary centres for compliance to the requirement. They think that the government has a role to play in compacting antimicrobial use and AMR problem. Awareness at all levels is needed. The extension officers have to visit farmers and advice accordingly starting from the construction of the animal houses structure, management and hygienic practices of the animal.

Waste management practices: All wastes are collected in a lump sum mode. They come from different sources therefore there is likelihood to be the source of AMR development and spread. From the household wastes are collected and sent to the collection point where the municipal vehicles do pass to collect. They were supposed to collect twice per week but sometimes they do it once per month therefore the entire wastes do scattered along the street.

Flooding: Very common along the basin in such a way that some of the affected families do vacate during rainy season. People along the basin do not collect their wastes; rather they just throw directly to the river. Additionally all the latrines are either connected or directed to the river in such a way that during rainy season they easily empty the effluents.

Location: Visegese Street Office
Interviewees: Livestock keepers, farmers, retired government officers and other community members

Eleven peoples aged from 21 – 60 were interviewed; one of them was above 60 years. They were all land owners in the area. One of them has lived for one year; the other for two years and the rest have been there for more than three years. All respondents have been working in animal farming for more than two years.

Animal farming: intensive farming with some few extensive farming in poultry for those who own land of one hector and above. Pig keepers are totally confided to avoid being seen by the Muslim worshipers. They normally feed them with maize bran bought from Gongolamboto, and food left overs. The poultry are feed by the commercially produced feeds but those keeping improved breeds; they sometimes let the animal scavenge in fenced areas.

Use of antimicrobials and accessibility: The broilers, layers and improves local breeds are given antimicrobials to protect them from diseases and also to treat them when they are sick. Pigs are also given drugs for protection and disease treatment. Although the

antimicrobials are easily accessible, they have to follow them to Gongolamboto as there are no reliable veterinary centres in Kisarawe town.

Diseases affecting pigs and poultry: Typhoid, Coryza, Coccidiosis, Respiratory diseases, Diarhoea, Newcastle and cannibalism are common problem with poultry. The pigs are affected by skin diseases, iron deficiency, worms, fever and diarrhoea with some rare cases of mastitis. Disease frequency is throughout but while some farmers see the problem during rainy season, other complains to be more severe during dry season.

Diagnosis and treatment. Disease diagnosis is based on clinical signs and symptoms. They are experienced enough to identify the sick animals and conduct treatment. However, they sometimes end up misdiagnosing and when this happen they call a private or government veterinary doctor for treatment. In some rare cases when treatment fails they have to send sample of sick animals to Dar es Salaam for diagnosis. For pigs when all measures fails or they see no signs of recovery they slaughter the animal but before consumption they call the veterinary for inspection.

Drugs used: In poultry they mostly use OTC, CTC, Trimazine, Tetracycline, Neomycine and ESB3. The human drugs such as Tetracycline, Metronidazole, Asprin, Ampicilin, and Ampiclox are used since they are cheap and readily available. In pigs they use Sulphur, Penstrep, Tylosin and Ivermectine.

Extension officers: There is a government livestock officer who visits them regularly. He has been of great help and contributes to reduction of disease frequency. The problem is he has no drugs, so once they were advised they have to go to Gongolamboto to purchase. They would wish the government officials to be allowed to carry drugs or have drugs with them because this might simplify their work. They are not aware of the laws and government regulation on antimicrobial sale as they have never been experienced difficulties during purchasing.

Waste management: Most of them had large areas to dispose the wastes although they are required by the district to collect and put in a collection point for the vehicle to pass. This has never been a problem because even if the vehicle does not pass they discard them in their small garden or give them to their animals. The problem is the payment that they have to make as a waste collection fees. The wastes are not sorted therefore all the wastes are disposed as they are.

Flooding: This is the area where the river originates. There is no much human activities and the valley is not that much occupied by human settlement. There is no flooding and agriculture activities along the valley are practiced to a small extent.
